# Discovery of electromagnetic polarization in Asian rice wine deterioration process and its applications

**DOI:** 10.1371/journal.pone.0302983

**Published:** 2024-06-20

**Authors:** Weijia Zhang, Xuejing Cao, Xue Cheng, Dongqin Sun, Tianfang Wei, Zebo Fang, Jiaju Li, Feiyu Chen, Xinghua Liu, Zhijian Cai, Chi Shen

**Affiliations:** 1 Department of Mathematical and Information Sciences, Shaoxing University, Shaoxing, China; 2 Institute of Artificial Intelligence, Shaoxing University, Shaoxing, China; 3 Visiting Scholar, Department of AOP Physics, University of Oxford, Oxford, United Kingdom; 4 National Engineering Research Center for Chinese Rice Wine (Branch Center), Shaoxing University, Shaoxing, China; 5 School of Life Sciences, Shaoxing University, Shaoxing, China; 6 Yingfu Tech Group Co. Ltd, Hong Kong, China; 7 Oxford Industrial Holding Group, Hong Kong, China; Center for Research and Technology Transfer, VIET NAM

## Abstract

Rice wine, known as yellow wine in China and Japan, possesses considerable nutritional value and holds significant global influence. This study addresses the challenge of preserving rice wine, which is prone to rancidity due to its low alcohol content. Conventional storage techniques employing pottery jars often result in substantial spoilage losses. Through rigorous investigation, this research identifies a polarization phenomenon exhibited by degraded rice wine when subjected to high-frequency microwaves(>60GHz), presenting a pioneering method for detecting spoilage, even within sealed containers. Employing a multi-channel microwave radar apparatus, the study delves into the susceptibility of rice wine to electromagnetic waves across various frequencies, uncovering pronounced polarization traits in deteriorated samples within the E-band microwave spectrum. Furthermore, lab-controlled simulations elucidate a direct correlation between physicochemical alterations and high-frequency Radar Cross Section (RCS) signals during the wine’s deterioration process. A novel six-membered Hydrated Cluster hypothesis is proposed, offering insights into the molecular mechanisms underlying this phenomenon. Additionally, dielectric property assessments conducted using vector network analyzers (VNA) reveal noteworthy enhancements in the dielectric constant of deteriorated rice wine, particularly within the high-frequency domain, thereby augmenting detectability. These findings carry implications for refining rice wine preservation techniques and contribute to the advancement of non-destructive testing technologies, enabling the detection of rice wine deterioration or indications thereof, even within sealed vessels.

## 1. Introduction

### 1.1. Industrial value, storage process, and easy deterioration of rice wine

Rice wine, also referred to as yellow wine in China and Japan, boasts a brewing legacy spanning nearly 4000 years in Asia, positioning it among the world’s oldest alcoholic beverages, alongside beer and grape wine, collectively recognized as the three ancient wines [1]. Presently, Chinese rice wine maintains an annual production stability within the range of 2.5 to 3.5 million kiloliters. In contrast, global wine production stands at approximately 25 million kiloliters, highlighting that Chinese rice wine output constitutes 1/10 of the worldwide wine production. The significance of this study lies in its potential to substantially reduce production costs for rice wine.

Rice wine is esteemed for its nutritional value. Crafted from wheat and various grains as primary ingredients, its production involves the utilization of Wheat Qu, Rice Qu, or alcoholic medicine as saccharifying agents. Through a meticulous process encompassing steps such as steaming rice, yeast fermentation, wine distillation, and storage [2, 3], rice wine achieves its distinctive flavor profile and aromatic characteristics.

Rice wine is renowned for its opulent bouquet and distinctive flavor profile, attributes stemming from the intricate interplay of diverse compounds synthesized throughout the fermentation process, encompassing esters, alcohols, aldehydes, acids, carbonyl compounds, and phenols. Notably, adherence to traditional consumption practices persists in China, Japan and other nations, underscoring the enduring cultural significance of this libation [4].

**Fig 1:**
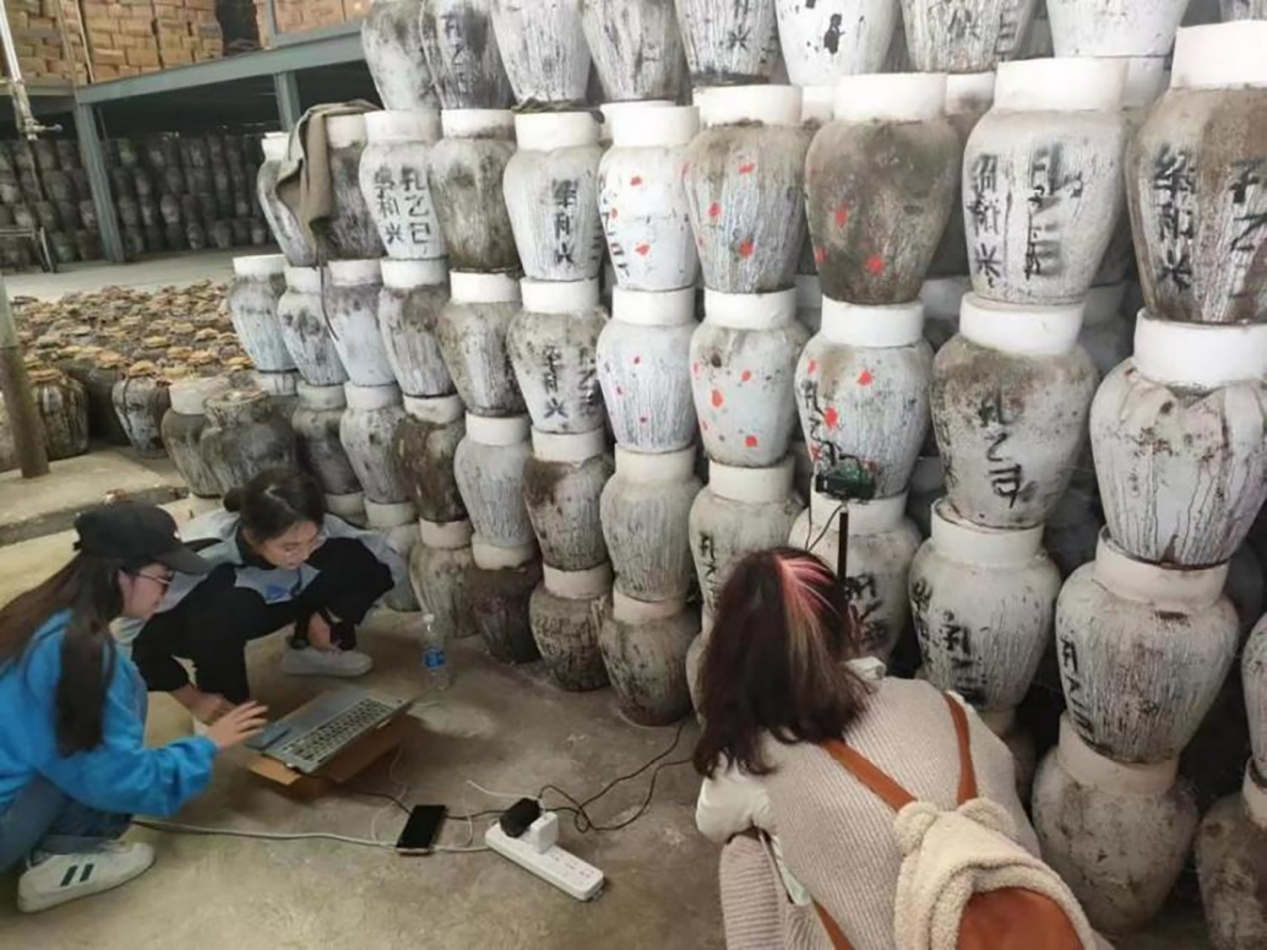
Captured photo of China’s rice wine storage at Shaoxing Wine Distillery during our team’s experimental work.

In both China and Japan, the traditional storage vessels for hand-brewed rice wine encompass a variety of materials, including pottery jars, carbon-steel tanks, and stainless-steel tanks. As shown in Fig 1, an example of rice wine storage at Shaoxing Wine Distillery in China. Prior to sealing the rice wine within these containers, it undergoes a rigorous high-temperature decoction process, effectively neutralizing the majority of aerobic microorganisms and enzymes. Subsequently, the hot wine is promptly transferred into aging pottery jars, featuring a clay cover surface, and tightly sealed using lotus leaves, bamboo shells, or similar materials. Notably, the utilization of pottery jars is favored for its exceptional permeability, facilitating accelerated redox and esterification reactions during the aging phase. It is essential to recognize that not all rice wines are amenable to prolonged aging, with certain varieties suitable for storage periods ranging from three to five years. Long-term storage viability is contingent upon the utilization of premium ingredients, masterful craftsmanship by the winemaker, and fortuitous contributing factors [5].

Nevertheless, the preservation of traditional rice wine poses a challenge due to its inherent susceptibility to souring, attributed to its relatively low alcohol content (usually <20%).

Hence, souring phenomena are frequently encountered during the storage of rice wine. Presently, rice wine enterprises predominantly derive their revenue from aged rice wine. Over a 10-year span, the average deterioration rate exceeds 20%, thus emerging as the primary cost-impacting factor within the rice wine industry as a whole.

The rancidity of rice wine primarily manifests as acetic and lactic acid rancidity. Acetic acid rancidity is prevalent in low-alcohol and aerobic conditions, often stemming from compromised or inadequately sealed wine jars [6, 7]. While disruptive, it poses less harm to the standard storage of rice wine. In contrast, lactic acid rancidity emerges as the predominant cause of deterioration during the aging process of rice wine in a closed environment. Notably, researchers have isolated Lactobacillus fructivorans and Lactobacillus acidophilus from sour rice wine. These strains exhibit high alcohol tolerance and thrive in acidic environments, contributing significantly to the occurrence of lactic acid rancidity.

In the assessment of rice spoilage, the conventional method necessitates the opening of the storage vessel for tactile examination and taste evaluation. Consequently, rice wine aged over decades can solely undergo sampling and analysis upon unsealing the container. Nonetheless, the unveiling of the vessel marks the conclusion of the entire rice wine aging process. In the event of souring, rot, or contamination, the entirety of the rice wine batch becomes unsalvageable, thereby diminishing the overall yield of the rice wine sector and consuming significant storage capacity, thereby augmenting production expenses considerably.

### 1.2. Chemical changes of rice wine during fermentation process

Consequently, the examination of the physico chemical attributes of degraded rice wine has garnered attention from previous researchers. Based on extant research, it has been observed that the alcohol content, sugar concentration, acidity, and pH levels of rice wine tend to decline with prolonged aging. Conversely, the levels of amino acid nitrogen and non-sugar solids exhibit varying degrees of augmentation. Ester concentrations reach their zenith at approximately five years before experiencing a modest decline thereafter, ultimately stabilizing [6–8].

Typically, substandard rice wine exhibits lower total sugar and alcohol levels compared to its unspoiled counterpart, accompanied by an elevated total acidity [9].

In Table 1, it is observed that the ethyl content experiences an increase after five years followed by a decrease after eight years, a pattern mirrored by ethyl hexanoate, octanoate, caprate, and the overall content. One plausible explanation for this phenomenon is that rice wine undergoes a sequence of chemical transformations during the initial phase of storage, predominantly characterized by reactions between acids and alcohols, culminating in the generation of ester compounds. As the storage duration progresses, it is anticipated that these ester compounds, alongside associated alcohol constituents, will gradually volatilize, thereby resulting in a downward trajectory in their respective contents.

**Table 1:** Major physical and chemical indicators and ester content of rice wine during storage [9].

	New wine	1 year old wine	3 years old wine	5 years old wine	8 years old wine
Alcohol content(%Vol)	16.83±0.20	16.42±0.22	15.80±0.13	15.41±0.21	15.20±0.15
Sugar(g/L)	24.22±0.33	23.61±0.45	22.61±0.28	21.63±0.44	21.14±0.26
Acid (g/L)	4.96±0.14	4.89±0.20	4.81±0.31	4.75±0.27	4.58±0.39
pH	4.21±0.00	4.13±0.01	4.08±0.02	3.92±0.01	3.88±0.01
α- Amino acid nitrogen (g/L)	0.79±0.06	0.80±0.05	0.80±0.03	0.83±0.09	0.86±0.07
Non-sugar solids (g/L)	22.66±0.47	21.76±1.25	25.78±1.70	27.77±1.25	32.56±0.82
Ethyl acetate (mg/L)	12.70±1.26	14.69±1.01	22.16±0.82	25.06±2.08	26.32±0.65
Ethyl lactate(mg/L)	16.27±0.83	18.48±1.32	21.50±2.65	23.07±0.74	21.07±0.26
Ethyl hexanoate (mg/L)	0.26±0.06	1.35±0.20	2.01±0.36	3.06±0.18	3.65±0.62
Ethyl octanoate (mg/L)	0.52±0.03	1.43±0.22	2.32±0.22	3.68±0.34	3.14±0.28
Ethyl caprate (mg/L)	0.21±0.04	0.78±0.16	1.01±0.26	0.89±0.14	0.84±0.08
Total ester content (mg/L)	29.96±2.22	36.73±2.91	49.00±4.31	55.76±3.48	55.02±1.89

Previous studies regarding the degradation of rice wine have predominantly centered on the examination of its chemical composition and observable physical attributes, yielding notable advancements in this domain [10, 11]. Nevertheless, a notable gap persists in the literature concerning the potential interaction between rice wine and electromagnetic waves. Given that rice wine is conventionally stored in pottery jars, characterized by their imperviousness to infrared radiation, this aspect remains largely unexplored.

Instead of infrared radiation, our research team utilized multi-channel microwave technology for detection, yielding notable results [12, 13]. Additionally, we successfully employed microwave technology for sub-surface machine vision, demonstrating commendable outcomes [14]. Notably, microwave, with its longer wavelength compared to infrared, proved advantageous for penetrating several centimeters or even meters into materials [15–19]. Given its ability to easily penetrate pottery jars storing rice wine, our team embarked on an innovative experiment using multi-channel microwave equipment. This endeavor aimed to investigate the sensitivity of rice wine to electromagnetic waves of varying frequencies throughout its aging and deterioration process.

This research aims to delve into the aging-related deterioration of rice wine, specifically exploring the distinctive polarization characteristics and electromagnetic alterations observed in deteriorated samples when exposed to specific frequencies of microwave electromagnetic waves.

The experiments unveiled a notable trend wherein deteriorated rice wine samples exhibited markedly heightened polarization characteristics in response to a specific high-frequency electromagnetic wave (specifically, the E-band microwave electromagnetic wave within the 60–70 GHz frequency range), contrasting with the good-quality rice wine samples. The research team conducted multiple corroborative experiments across diverse groups, thereby establishing this phenomenon for the first time.

This previously unreported phenomenon holds promise as a potential marker for assessing rice wine quality. Furthermore, it could serve as a valuable tool for detecting signs of rice wine deterioration, even when stored in sealed containers, and potentially for monitoring other fermentation processes that yield acidic byproducts.

## 2. Methods and experiments

### 2.1. High frequency RCS differences found in spoiled rice wine

The samples utilized in our investigation were sourced directly from the distilleries and meticulously assessed by professional technicians to categorize their quality status as normal, mildly deteriorated, or severely deteriorated. Each sample underwent a comprehensive evaluation process to ascertain the extent of deterioration. Severely deteriorated samples exhibited pronounced acidity levels, visible microbial proliferation, and emitted off-putting odors discernible by trained sensory panelists. Conversely, mildly deteriorated samples displayed elevated acidity levels, a sour taste lacking the typical flavor profile, yet devoid of visible microbial growth. Prior to sample provision, the distillery conducted extensive physical and chemical analyses alongside sensory assessments by trained experts, furnishing sample data to validate their quality classification.

**Fig 2:**
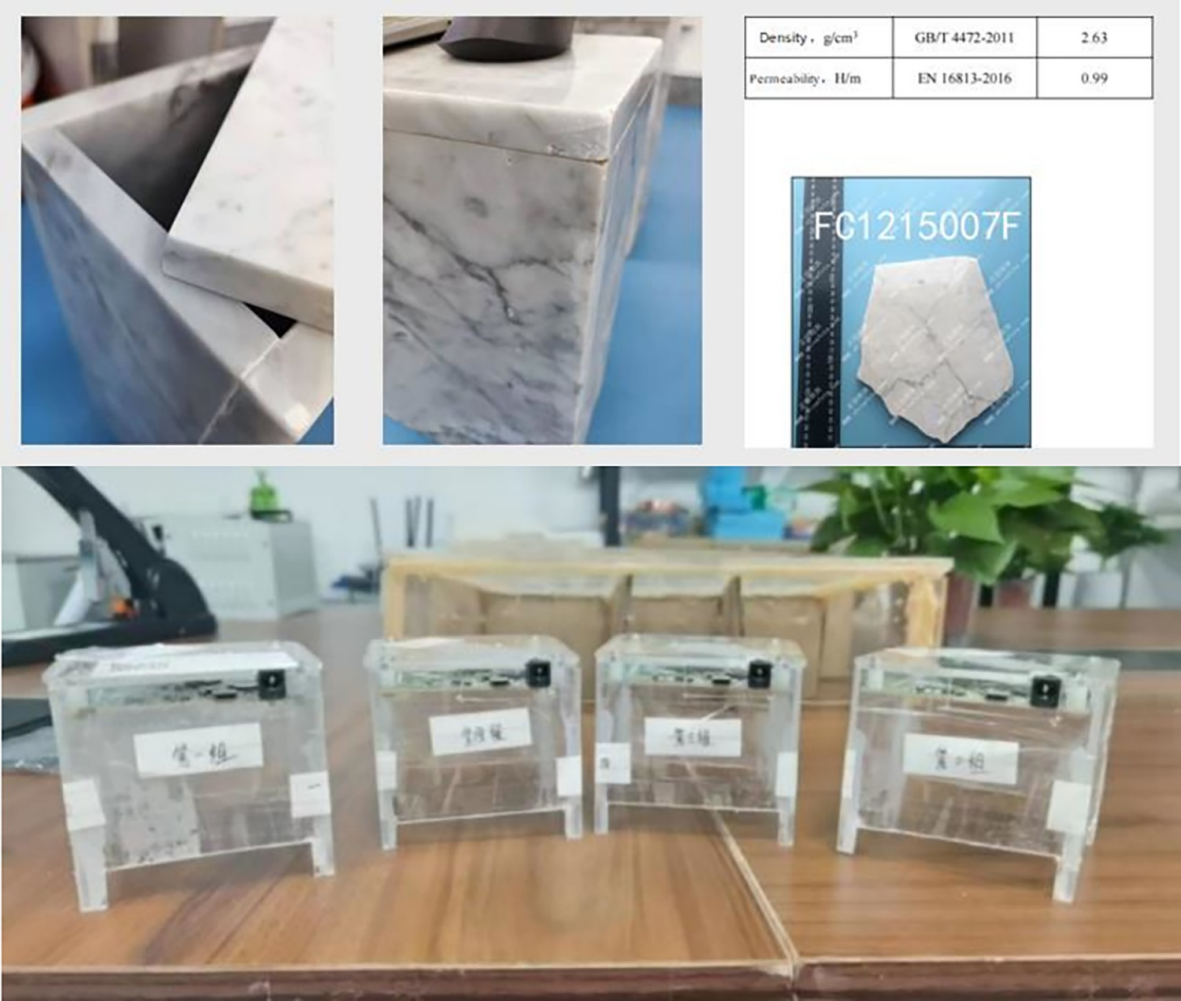
(a): Quality inspection machine measures two wine jars with customized parameters, observing differences in echo imaging value curves. (b): Up to 69GHz high-frequency microwave detector.

We initially devised an experiment targeting ordinary and mildly deteriorated rice wines, both sourced from the Chinese National Yellow Wine Engineering Experimental Center. Employing identical containers with known dielectric properties, as shown in Fig 2(A) and 2(B), (two custom-made marble containers with a measured density of 2.63 g/cm^3^ and a magnetic permeability of 0.99 H/m), we utilized a microwave radar probe to vertically emit electromagnetic waves into the container and capture their echo signal. The radar equipment offered a range of frequencies, including 300 MHz, 2.2 GHz, 6.8 GHz–8 GHz, 29 GHz, and 60 GHz–70 GHz.

The aforementioned microwaves demonstrated efficient penetration through the structure of the 1 cm-thick marble wine container, reflecting upon encountering the interface between the wine and the container. Both wine containers exhibited identical outward appearances. One container held 2014 GYLS rice wine of standard quality, produced and sealed in 2014, boasting a pH value of 4.4. Conversely, the other container housed samples from the same 2014 batch, yet classified as spoiled by the Chinese National Rice Wine Engineering Experimental Center due to a pH value of 3.7, and a sour taste.

The experimenters meticulously observed and meticulously documented the imaging data pertaining to the microwave radar cross-sectional area (referred to as Radar Cross Section, or RCS).

Prior to conducting measurements, the radar equipment was powered on for an extended duration to ascertain its stable operational state post-warm-up. Throughout this period, the radar temperature remained constant, mitigating any potential drift in RCS values attributed to temperature fluctuations. This standardized protocol was consistently implemented for each experimental group.

At lower frequencies, no discernible distinctions were observed. Nevertheless, as the frequency progressively surpassed 60 GHz, noteworthy variations in the measured Radar Cross Section (RCS) values between the two groups became evident. Specifically, the 69GHz RCS values for the substandard rice wine group were approximately 10% higher compared to those of the superior rice wine group.

This discrepancy persisted in all the repeated tests (>20), and even after swapping and exchanging the wine containers, effectively eliminating the possibility that variations stemmed from the specific wine containers utilized in the experiments.

For a given observed entity, the RCS is contingent upon factors such as the object’s surface topology, the frequency of the microwave, and the polarization of the observed object. Essentially, the RCS of a target represents the hypothetical area visible to radar systems. It denotes the area that, when uniformly scattered in all directions, yields a radar echo equivalent to that of the target. This relationship can be expressed mathematically as follows:

$$\sigma =\underset{R\to \infty }{\lim}\;4\pi R^{2}\frac{{\left|E_{S}\right|}^{2}}{{\left|E_{i}\right|}^{2}}$$ (Eq 1)

where:*R* = distance between radar and target, *E*_*s*_ = scattered field strength at radar, *E*_*i*_ = incident field strength at target

As elucidated in Merrill I. Skolnik’s seminal work, "Introduction to Radar Systems" [15], the radar cross-sectional area undergoes alterations contingent upon the angle, frequency, and polarization. When maintaining consistency in the first two parameters, any variation in RCS delineates shifts in the polarization characteristics.

With the angle and frequency remaining constant, and given the observed disparities in RCS values, we postulate a potential augmentation in the polarization properties of the substandard rice wine at microwave frequencies surpassing 60 GHz.

### 2.2. RCS measurements validation on rice wine and different liquids with known polarity sequences

**Fig 3:**
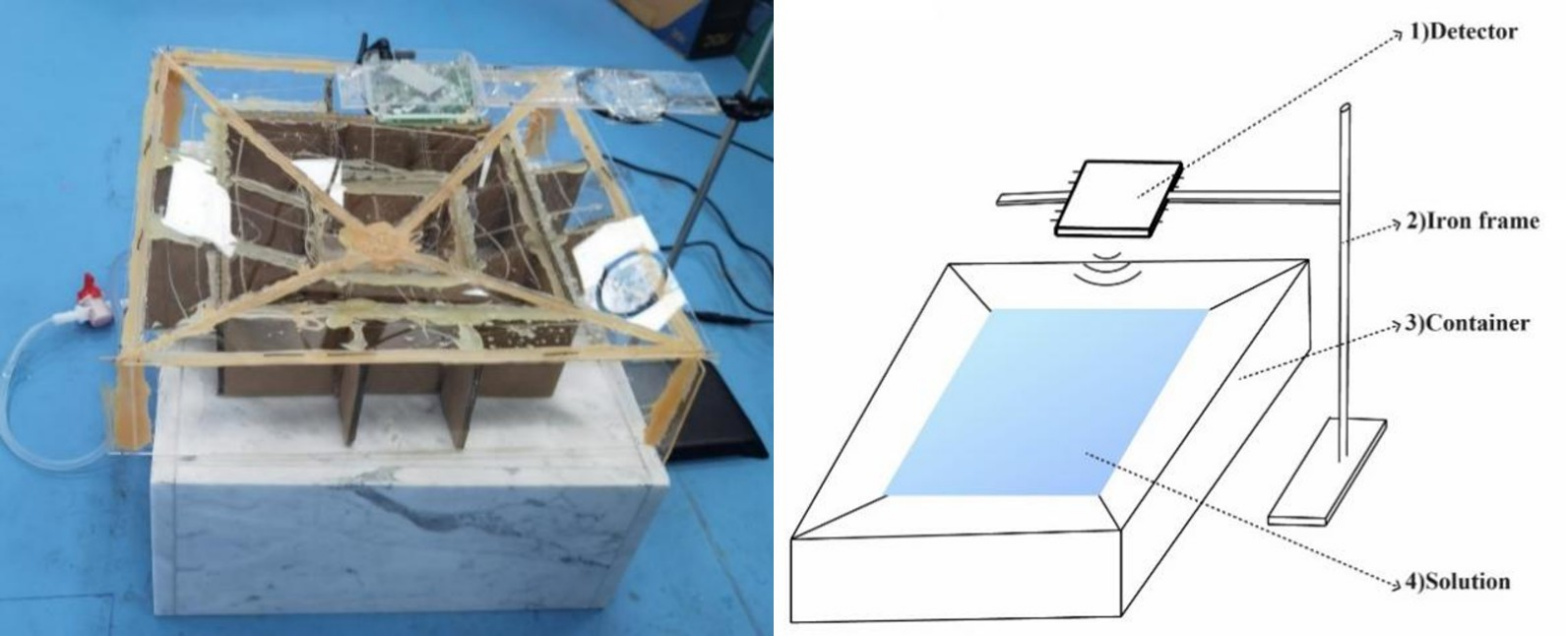
Specialized experimental device with fixed radar and a siphon to completely remove liquid after each test. Tested liquids: distilled water, pure ethanol, formamide, kerosene, normal rice wine, and bad rice wine.

Following the revelation of this phenomenon wherein inferior rice wine exhibits a significantly elevated echo RCS value above 60GHz, our subsequent inquiry aimed to corroborate the electromagnetic polarization of such subpar rice wine. To achieve this objective, we orchestrated a secondary set of experiments encompassing a broader array of severely degraded rice wine samples, normal rice wine samples from the same production batch, alongside various liquids of established polarity. Within this experimental paradigm, a high-frequency millimeter-wave radar was meticulously positioned directly above the liquid interface within the vessel, as shown in Fig 3, sending 69 GHz high-frequency E-band microwaves towards the liquid’s surface below, and subsequently capturing their echoes.

**Fig 4:**
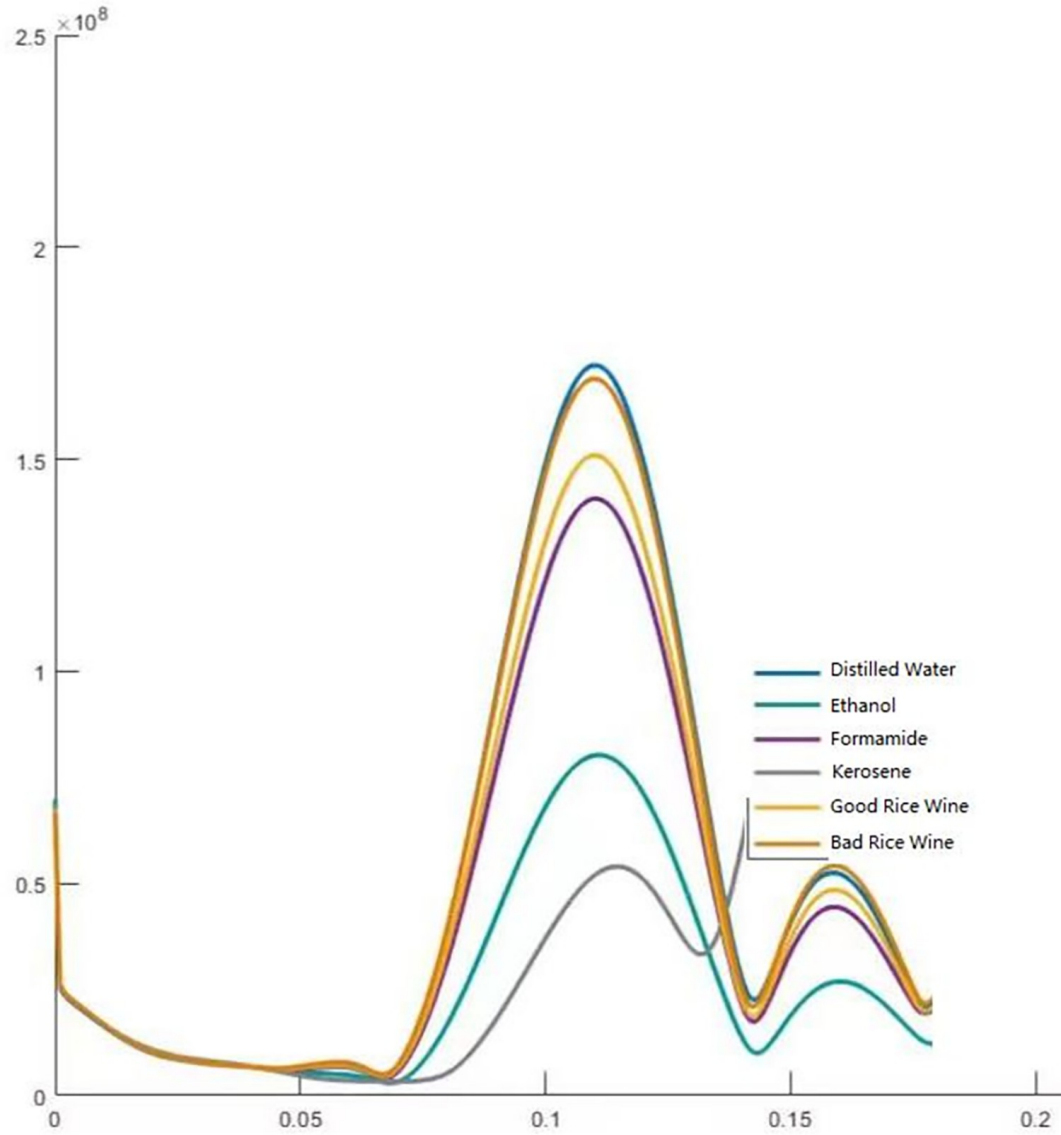
The vertical target is the imaging value (*Y*) after radar data processing (proportional to RCS [15], validated in supporting materials S1 Appendix), and the horizontal coordinate is the distance between the liquid surface and the microwave radar (in meters).

Fig 4 shows the result of our second experimental measurement.

In Fig 4, each curve corresponds to a specific type of liquid, with the peak value indicative of its RCS or polarity. Analysis of the data graph reveals that the RCS or polarity exhibited by severely degraded rice wine is only marginally lower than that of distilled water at 69 GHz, yet notably surpasses that of conventionally produced rice wine of standard quality. It’s worth noting that measurements for each liquid were derived from a consistent set of 20 samples, ensuring the experiment’s robust repeatability.

### 2.3. Experiments conducted across pottery jars as real industrial conditions

**Fig 5:**
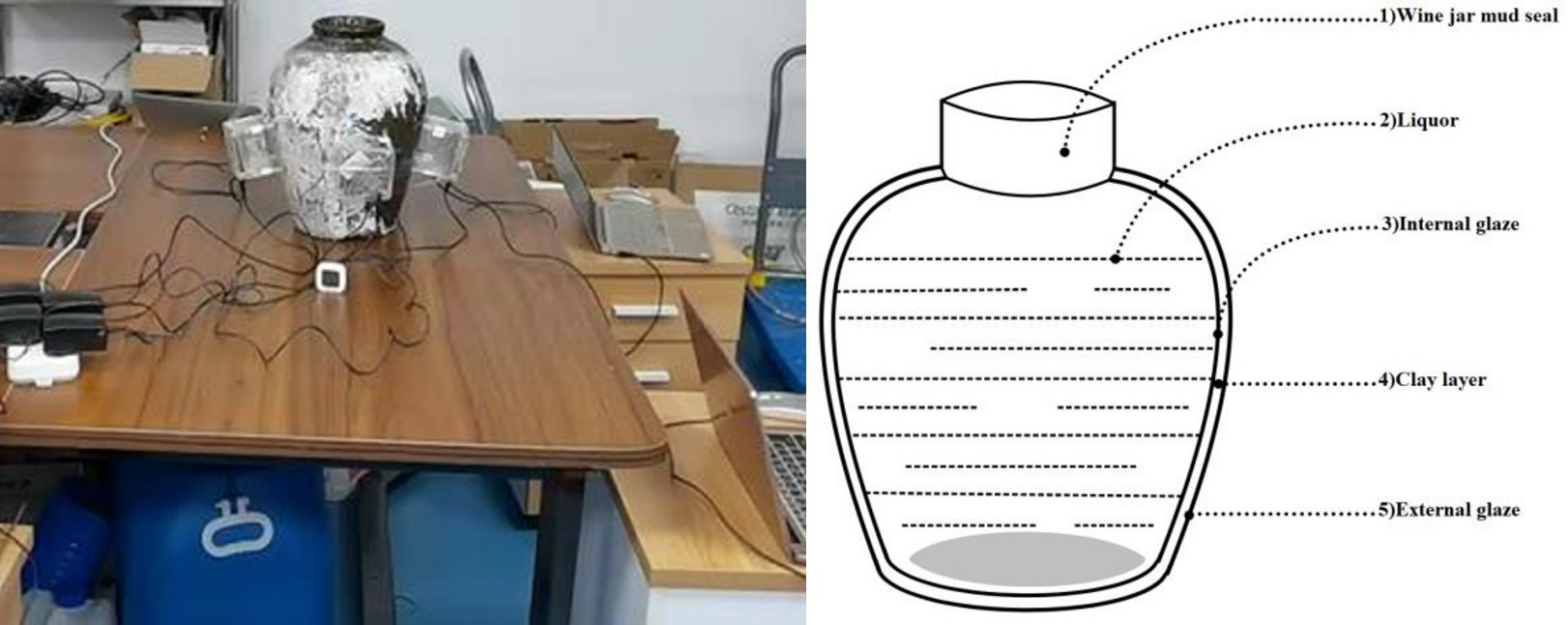
Photo of our testing process. Full experiment video uploaded at www.geoscience.ac.cn/usx/microwave/ricewine.zip.

In order to ascertain the practical industrial applicability of this revelation and its potential to permeate pottery vessels for quality monitoring across the pottery jars, a third set of experiments was undertaken. In this experimental series, we positioned four microwave radar devices around an pottery wine jar, each separated by 90 degrees,as shown in Fig 5. Subsequently, four independent observations were conducted concurrently, each facilitated by a dedicated computer system and overseen by a distinct supervisory experimenter.

In this experiment, the frequency of 69 GHz was employed. Once the radar attained a stable operational state, measurements commenced. Initially, the empty wine jar was measured to establish the baseline RCS value of the radar. Subsequently, water was added to obtain the corresponding RCS value. Following this, a siphon device was utilized to pump all the water, and after thorough drying, standard quality rice wine (specifically, Jia Fan from 2014, with a pH value of 4.4) was added to measure the radar’s RCS value. The process was repeated with mildly deteriorated rice wine (Jia Fan from 2014, spoiled, pH 3.7). Given the findings from the preceding experiments, where milder deterioration exhibited lower polarity than severe deterioration, the ability to detect mild deterioration suggests the feasibility of detecting severe deterioration.

This database is publicly accessible, enabling anyone to download and peruse our experimental procedures.

**Fig 6:**
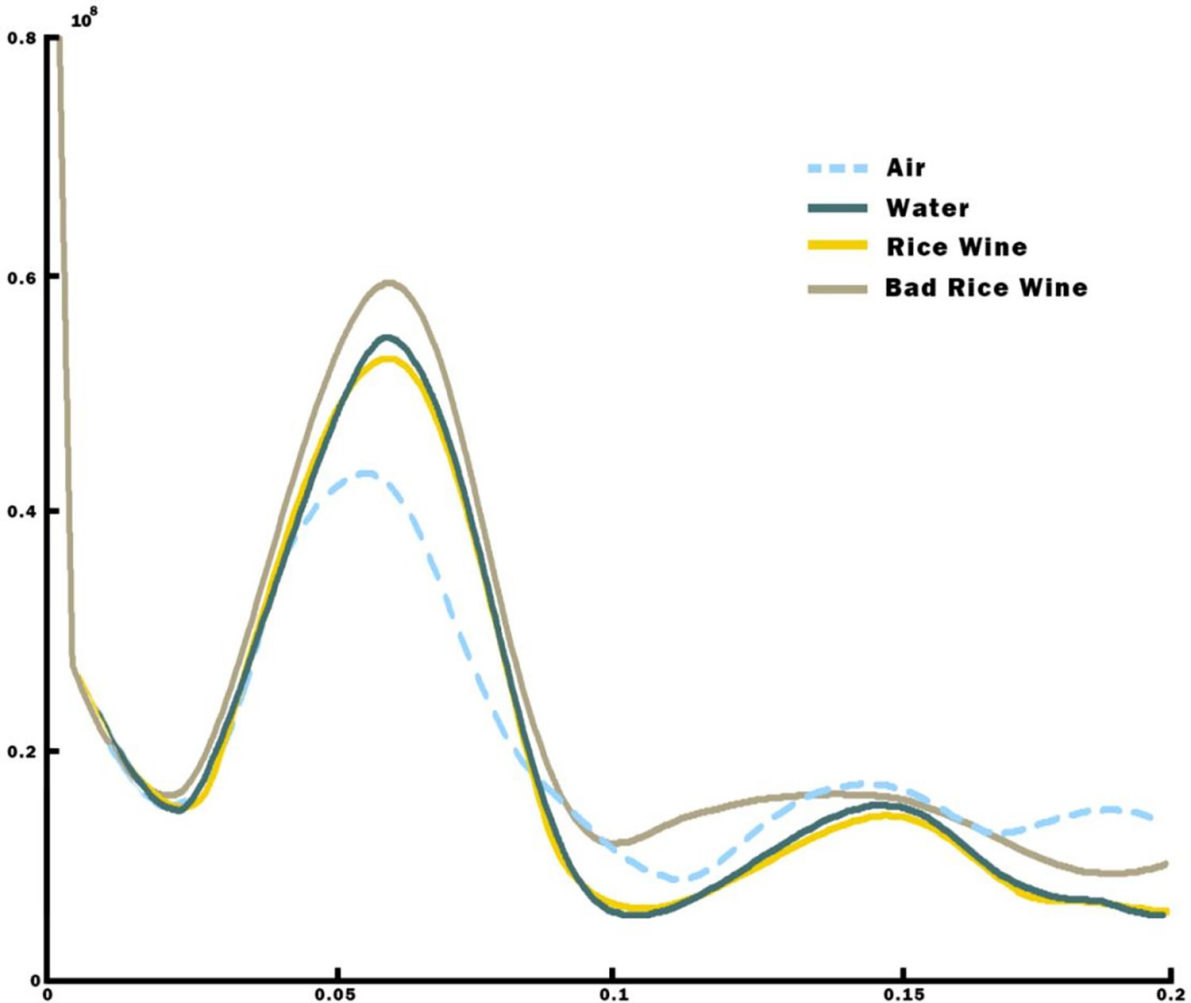
The imaging value (Y) after radar data processing, while the horizontal value (X) is the distance between the liquid surface and the radar, in meters.

As shown in Fig 6, the observed values of air by each radar device consistently ranked as the lowest, succeeded by those of the standard quality rice wine, water, and finally, the inferior rice wine registering the highest value. Consequently, the inferior rice wine utilized in this experiment exhibited a more pronounced polarization reaction even compared to water.

In summary, this set of experiments confirmed the presence of the electromagnetic polarization phenomenon in rice wine following deterioration.

Given that microwaves can penetrate rice wine jars, prolonged observation at a fixed position by millimeter-wave radars could enable the monitoring of RCS changes, thus indicating rice wine deterioration without necessitating container opening. Consequently, such polarization phenomenon harbors promising industrial applications.

## 3. Validation

### 3.1. Validation: 8GHz/65GHz/69GHz dielectric properties of rice wine before and after deterioration measured with vector network analyzer (VNA)

To validate the discovery, we employed a vector network analyzer (KeySight M9375A PXIe VNA) to assess if there are increases in relative permittivity following deterioration. The VNA facilitates the transmission of a generated microwave signal source to the open wave guide transmitting antenna through a corresponding coaxial connecting cable, utilizing the tested liquid substance as the microwave transmission medium. Interactions between the microwave and the liquid substance occur as the electromagnetic wave traverses through it. The open wave guide receiving antenna captures the transmitted signal, which is then conveyed to the VNA, equipped with an internal computer, through the coaxial connecting cable. Subsequently, the received microwave signal undergoes analysis and processing to ascertain the dielectric constant of the liquid samples.

**Fig 7:**
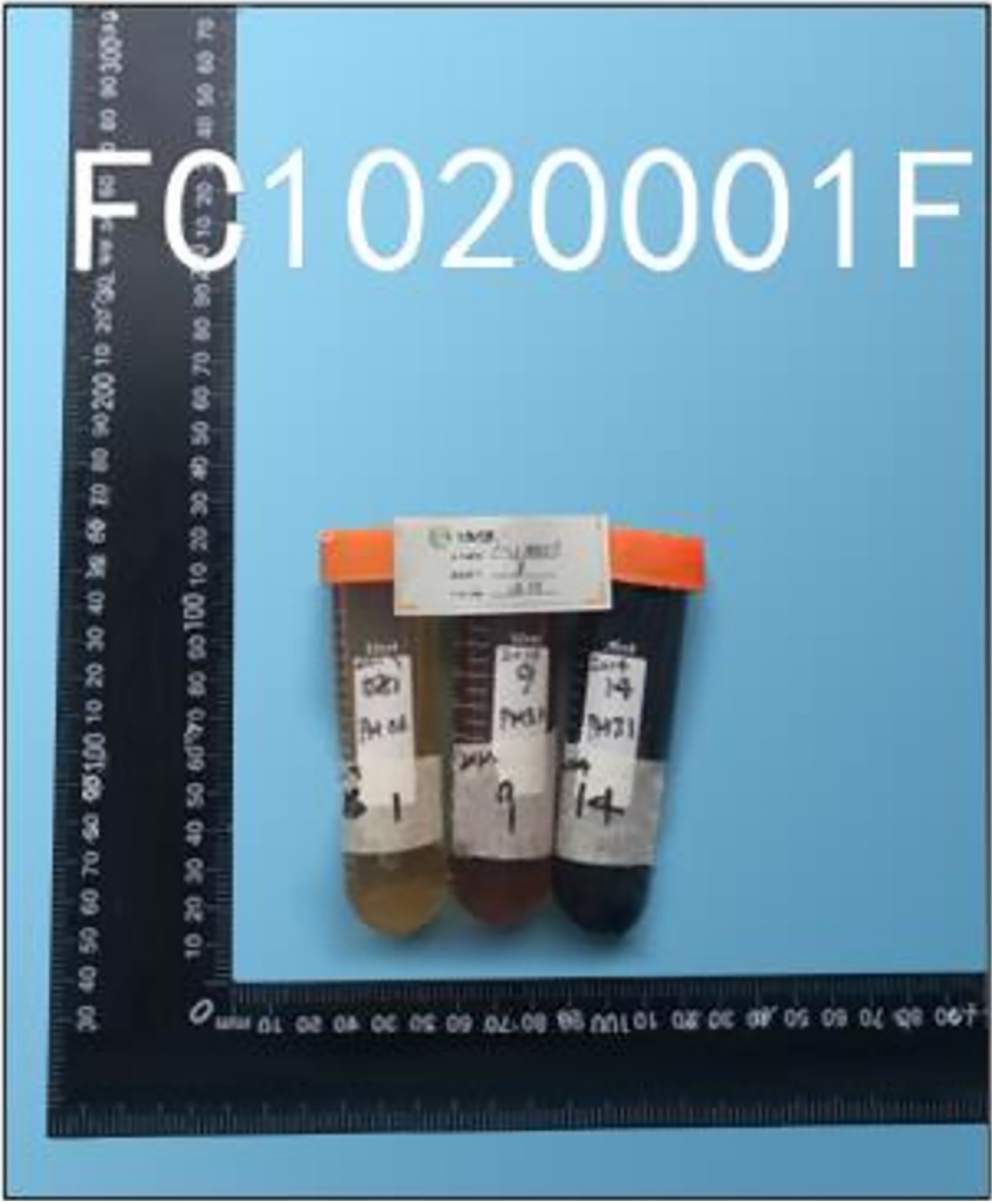
Samples from China’s National Rice Wine Engineering Center (labeled 2019–1, 2014–9, and 2014–14) undergoing dielectric constant tests by detection personnel. Results annotated in Table 2.

**Fig 8:**
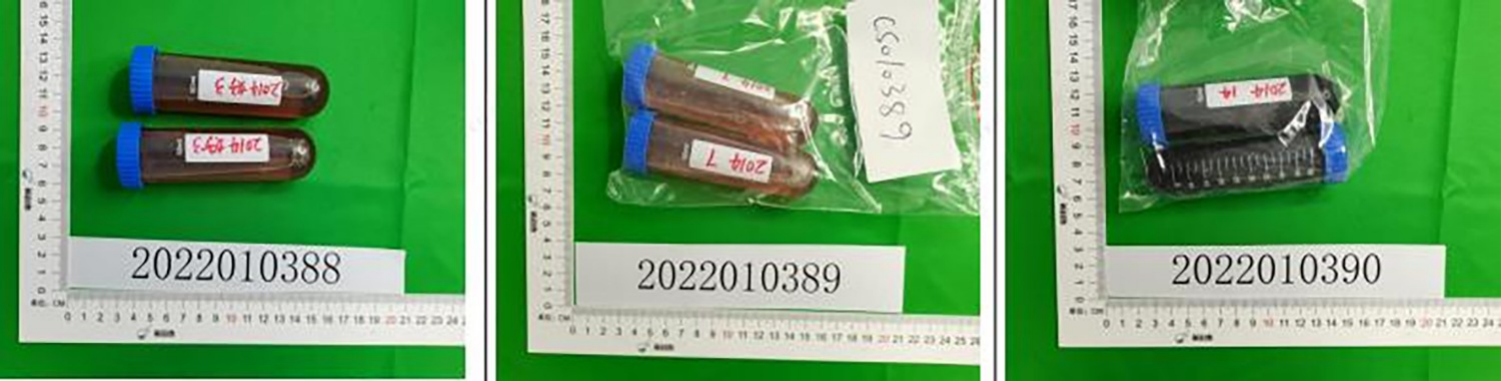
Samples 2019–1, 2014–9, and 2014–14 from KuaiJiShan undergo dielectric constant testing by examining personnel. Report available at www.feifan-sz.cn with report or validation number 44577035.

Through the assessment of the dielectric constant of rice wine (samples shown in Figs 7 and 8 across various frequency bands, a distinct variation in dielectric constant emerged between spoiled and non-spoiled rice wines, particularly notable in high-frequency bands.

As depicted in Table 2, the dielectric constant contrast between deteriorated and non-deteriorated rice wines at 69GHz surpasses 60%, indicating a pronounced significance in polarity divergence under high-frequency microwaves beyond 69GHz. Conversely, this distinction only reaches less than 10% at 8GHz.

**Table 2:** Test result of the dielectric constant of rice wine (the test verification agency is Qingdao Zhengxin Testing and Analysis Co., Ltd., and the test verification report number is FC1020001F. Report can be checked at www.chinafcta.com with report number).

Sample Name	Measured Properties		Method Standard	Results
2019	Dielectric constant	8 GHz	GB/T 5654–2007	59.8817±1.9162
Good quality 1		69 GHz		5.0012±0.1885
2014	Dielectric constant	69 GHz	GB/T 5654–2007	4.6553±0.1955
Good quality 1				
2014 9	Dielectric constant	8 GHz	GB/T 5654–2007	61.4516±3.1340
Mild deterioration		69 GHz		8.0225±0.3771
2014 14	Dielectric constant	8 GHz	GB/T 5654–2007	67.4872±2.2271
Severe deterioration		69 GHz		9.1308±0.3287

This outcome underscores that in the low-frequency microwave spectrum, disparities in dielectric constants are less pronounced, whereas they become markedly discernible at higher frequencies. These differences are likely attributable to the sensitivity of the utilized equipment, offering insights into the observed phenomena within our experiments.

A conspicuous 60% distinction precludes the possibility of other potential errors. The heightened polarity of spoiled rice wine under high microwave frequencies is unequivocal. Furthermore, this discernible enhancement holds practical significance, offering potential industrial applications for non-invasive quality measurement of rice wine without the need to open its container.

To validate these findings, additional experiments were conducted using samples sourced from KuaiJiShan, another renowned rice wine producer. The expanded sample size accounted for various factors including distinct batches, manufacturers, and vessels. In this iteration, measurements of both dielectric constant and dielectric loss were performed concurrently. The outcomes are summarized in Table 3.

**Table 3:** Test result of the dielectric constant of the second batch rice wine (the test verification agency is SUZHOU FeiFan Testing and Analysis Co., Ltd., and the test verification report number FTS202201083).

Sample Name	Measured Properties	Method Standard	Results
2014	Dielectric constant at 65 GHz	GB/T 5654–2007	19.0949
Good quality3	Dielectric loss at 65 GHz		0.9311
2014	Dielectric constant at 65 GHz	GB/T 5654–2007	20.2501
Mild deterioration 7	Dielectric loss at 65 GHz		1.0281
2014 14	Dielectric constant at 65 GHz	GB/T 5654–2007	28.8231
Severe deterioration	Dielectric loss at 65 GHz		2.4756

The findings from the second batch of samples, as presented in Table 3, corroborate those of the initial batch outlined in Table 2. Notably, substantial discrepancies in relative permittivity, encompassing both dielectric constant and dielectric loss, are evident within the high-frequency (>60GHz) microwave spectrum.

### 3.2. Further validation of the results and correlation between 60GHz RCS values & acidic components

The principal chemical compositional alterations in rice wine before and after deterioration primarily revolve around changes in acidic molecules content [20]. For instance, the rapid fluctuation in total acid content during the pre-fermentation phase of rice wine, as detected through near-infrared spectroscopy [20], is notable. Additionally, lactic acid bacteria engage in esterification reactions with ethanol, leading to the decomposition of weakly polar ethanol and consequent elevation in the proportion of more polar substances, such as lactic acid.

Hence, the most immediate hypothesis to consider is that the high-frequency electromagnetic polarization effect we have uncovered may be linked to alterations in the liquid components, particularly the production of acidic substances, within yellow rice wine.

In order to substantiate the connection between the total acid content present in alcoholic beverages and the RCS values identified by radar, the research team devised an additional validation experiment. This endeavor sought to explore the correlation between the total acid content inherent in spoiled rice wine and the corresponding detection values obtained from radar equipment RCS. The objective was to scrutinize the interrelationship between the total acid molecular content of liquid components and the resultant RCS values gleaned from radar imaging.

#### (1) Preparations

The requisite materials for the experiment encompassed rice wine (Shanniang Brand, produced in 2019), standard sodium hydroxide titration solution, distilled water, acrylic cubic containers, a thermometer, high-frequency radar equipment, MS-H-Pro+ magnetic stirrer, pipette, Z-axis manual lift platform (HTZ-120), acidity meter, calipers, and ruler. In order to mitigate the potential influence of temperature variations on the experimental results obtained from the high-frequency radar equipment, the solution temperature was meticulously maintained at 26°C to uphold data accuracy.

#### (2) Procedures

Controlled deterioration was induced in rice wine through the addition of 1 gram of yeast to 500 milliliters of Shaoxing rice wine. Subsequently, the mixture was incubated in a constant temperature chamber set at 37°C.

Deterioration became evident, with the flavor exhibiting a sour profile, commencing on the third day of observation.

The analysis of total acidity data is conducted through the utilization of the following mathematical model:

$$X=\frac{(V_{1}-V_{2})\times C\times M}{\left(V_{3}\right)}$$ (Eq 2)

In the provided equation, X denotes the total acidity content within the sample, delineated in grams per liter (g/L). C represents the concentration of the standard sodium hydroxide titration solution, expressed in moles per liter (mol/L). The variable M signifies the numerical value of the molar mass of lactic acid, articulated in grams per mole (g/mol), with a defined value of 90. *V*_1_ corresponds to the volume of standard sodium hydroxide titration solution utilized during the titration of the sample, quantified in milliliters (mL), while *V*_2_ signifies the volume of standard sodium hydroxide titration solution employed during the blank test, also measured in milliliters (mL). Finally, *V*_3_ denotes the volume of the extracted sample, measured in milliliters (mL).

#### (3) Data collection and analysis

Adhering to the procedures delineated in the Chinese National Testing Standard GB/T 13662–2018 "Rice Wine," total acidity measurements were systematically executed to uphold the precision and replicability of the experiment.

During each acid measurement, 10 mL aliquot was carefully transferred into a 150 mL beaker, followed by the addition of 50 mL of degassed water. Subsequently, a magnetic stirring bar was introduced into the beaker, which was then positioned atop a magnetic stirrer and set into motion. Standard sodium hydroxide titrant was incrementally added into the beaker until the pH reached 8.20, indicating the equivalence point, at which juncture the volume of 0.1 mol/L standard sodium hydroxide titrant utilized was meticulously documented. Concomitantly, a control experiment employing an equivalent volume of degassed water was conducted, with the corresponding volume of titrant utilized being meticulously recorded.

Concurrently, the 60GHz RCS radar apparatus was employed to capture imaging data. Initially, the lifting platform’s elevation was consistently set to 4.8 cm for each measurement to ensure uniform radar height. Subsequently, the liquid level was meticulously adjusted to maintain a consistent horizontal alignment throughout the measurements. The region of interest selected for measurement was the interface between the yellow wine and air, positioned 5 cm above the base of the acrylic container (factoring in the 4 cm thickness of the acrylic plate). Data acquisition was conducted, and subsequent to recording and storage, total acidity was derived utilizing a mathematical model. Linear regression analysis, facilitated by Origin2021 software, was then employed to investigate the correlation between total acidity and radar imaging values.

All total acidity and measured RCS values, along with other data, are accessible in S2 Appendix. The average total acidity within the rice wine and deteriorated rice wine group peaked at 9.90 g/L, accompanied by a notably higher average radar-measured value.

Sample testing were did 2–3 times daily, with temperature adjustments preceding each test to maintain consistency. Each test session was spaced at least one hour apart. Testing spanned from July 9, 2023, to August 5, 2023, 4 data sets were excluded from analysis due to significant errors resulting from improper experimental procedures.

The experiment revealed a discernible positive correlation between total acidity and radar scattering characteristics. As depicted in Fig 7, both RCS and the total acidity within the rice wine solution demonstrated a consistent upward trajectory.

**Fig 9:**
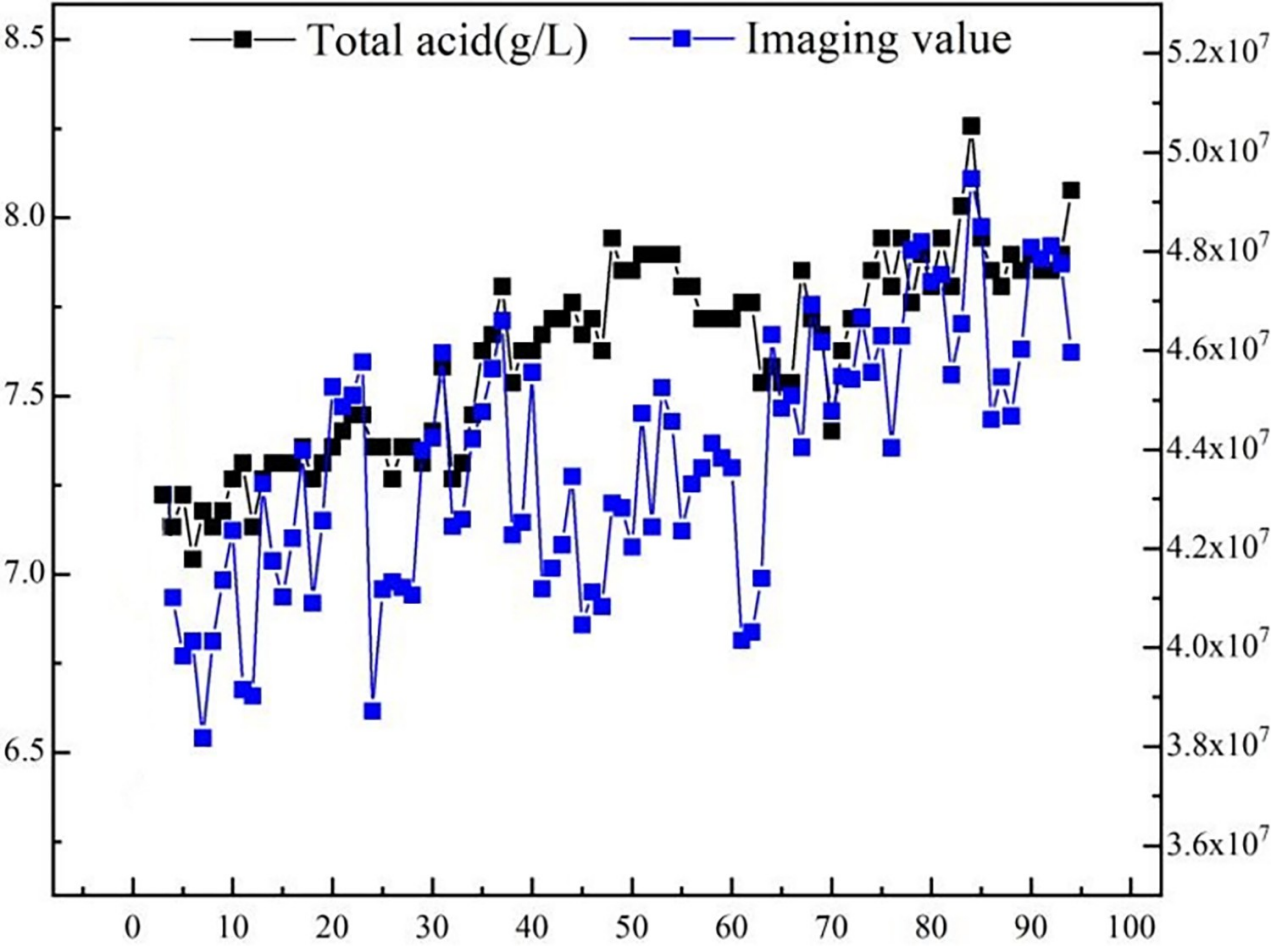
Horizontal axis represents 90 cumulative samples, left vertical axis shows rice wine total acidity (g/L, black line), and right vertical axis displays radar imaging values (proportional to RCS, blue line) with a clear positive correlation. Original data recorded in S2 Appendix.

The findings illustrated in Fig 9 indicate a pronounced positive correlation between total acidity and radar scattering characteristics when viewed holistically.

Drawing from the experimental findings outlined in this section, it is rational to propose that the high-frequency electromagnetic polarization effect observed in deteriorated rice wine is linked to the emergence of newly formed acidic components. In the subsequent section, a theoretical model alongside computer simulations will be presented to further elucidate these phenomena.

## 4. Discussion

This section of the study endeavors to explore the molecular polarization model of deteriorated rice wine under high-frequency microwaves in detail,propose a hydrated cluster model as an explanation for all above, and entail a quantitative examination of electronic molecular transitions and liquid polarization, culminating in the development of a theoretical framework via Gaussian Software computer simulation fitting.

RCS is known to exhibit a positive correlation with polarity, primarily because RCS directly mirrors the relative permittivity (ε), which in turn is closely associated with the polarity of molecules. Polar molecules like water have high permittivity (78), while less polar ones like cyclohexane have low permittivity (2).This relationship arises because ε reflects the mitigation of electrostatic effects induced by the medium. In the case of liquids comprised of polar molecules, the application of an electric field prompts molecular polarization. The resultant counteracting electric field generated by these polarized molecules partially offsets the external electric field, leading to a more rapid attenuation of electrostatic effects compared to that observed in a vacuum.

In section 3, the research team uncovered that the positive correlation between RCS and total acidity content suggests that as the total acidity increases, the polarity of the deteriorated rice wine solution intensifies.

This phenomenon can be explained from following aspects:

Firstly, ethanol and water constitute the primary components of rice wine prior to deterioration. It is well-established that pure water exhibits high polarity. Conversely, ethanol exhibits non polarity. Consequently, the polarity of rice wine primarily dictated by the proportion of water present. During the deterioration of rice wine, ethanol undergoes decomposition and concurrent increase in various polar acid forms thereby elevating the proportion of polar molecules.Notably, L. acidophilus stands out as the primary microorganism responsible for inducing the rancidity of rice wine, followed by L. fructose [21]. Both acid molecules and water molecules exhibit greater polarity than ethanol, consequently leading to an augmented radar Radar Cross Section (RCS) response. This observation aligns with the theory posited by Yu Derun [22] regarding empirical parameters of polarity and the proportional relationship between the dielectric constant of solvents and their polarity. Moreover, research by Zhao Donghui [23] and others affirms that, at the same temperature, the order of polarity for common solvents is water > acid > alcohol.

Secondly, recent spectroscopic investigations over the past decade [24] have revealed the formation of various ethanol hydrate clusters between liquid ethanol and water molecules. Prior to deterioration, of, rice wine, typically contains an alcohol content ranging from 14% to 20%, with water volume fractions ranging from 70% to 90%. Under these conditions, the hydration clusters predominantly consist of hydrogen bonds formed between water molecules and ethanol molecule hydroxyl groups, characterized as (H2O) m′(EtOH) n′, where n′<n. As the water volume content continues to rise, the hydrophilic hydration between (H2O)m clusters and ethanol molecule hydroxyl groups gradually approaches saturation. Consequently, the relative volume occupancy of water molecules in comparison to ethanol expands. Post-deterioration, water molecules further interact with the hydrophobic CH group of ethanol molecules, fostering the formation of additional C–H…O hydrogen bonds. Notably, as water content increases, the strength of hydrogen bonding intensifies, leading to a shift in the frequency of C–H stretching vibration towards the high-frequency band [25].

The emerging constituents, such as lactic acid and acetic acid, within deteriorated rice wine likely adopt analogous structural forms, leading to the formation of novel hydration clusters. Lactic acids, characterized by their polar nature, participate in the creation of fresh polar molecular clusters with water molecules. When the vibration frequency of a specific entity falls within the high-frequency spectrum, it can precipitate the phenomenon of electromagnetic response enhancement within that frequency range.

Thirdly, and most importantly, a mechanism we consider paramount involves the generation of newly formed lactic acid and acetic acid, which may amalgamate into novel molecular clusters with water molecules. This resultant configuration is anticipated to exhibit higher polarity compared to the initial ethanol–water molecule clusters, consequently amplifying the response of high-frequency radar RCS.

In addressing this aspect, we performed quantum mechanical calculations pertaining to the second point. In a seminal study conducted in 1988, Semmler et al. [26] employed Raman spectroscopy to examine the spectra of acetic acid aqueous solutions. Their findings pointed towards the prevalence of predominantly cyclic dimers and linear dimers within the acetic acid aqueous solution [26].

Following this, scholars posited that cyclic dimers exhibited instability in polar solutions. In 1990, Yamamoto et al. [27],Substantiating this notion. In 2003, Chocholousova et al. [28], highlighted that within a micro-water environment, water molecules engage with dimers. This interaction leads to the association of water molecules with acetic acid molecules through new hydrogen bonding, resulting in the formation of water-separated dimer structures (WSD).

**Fig 10:**
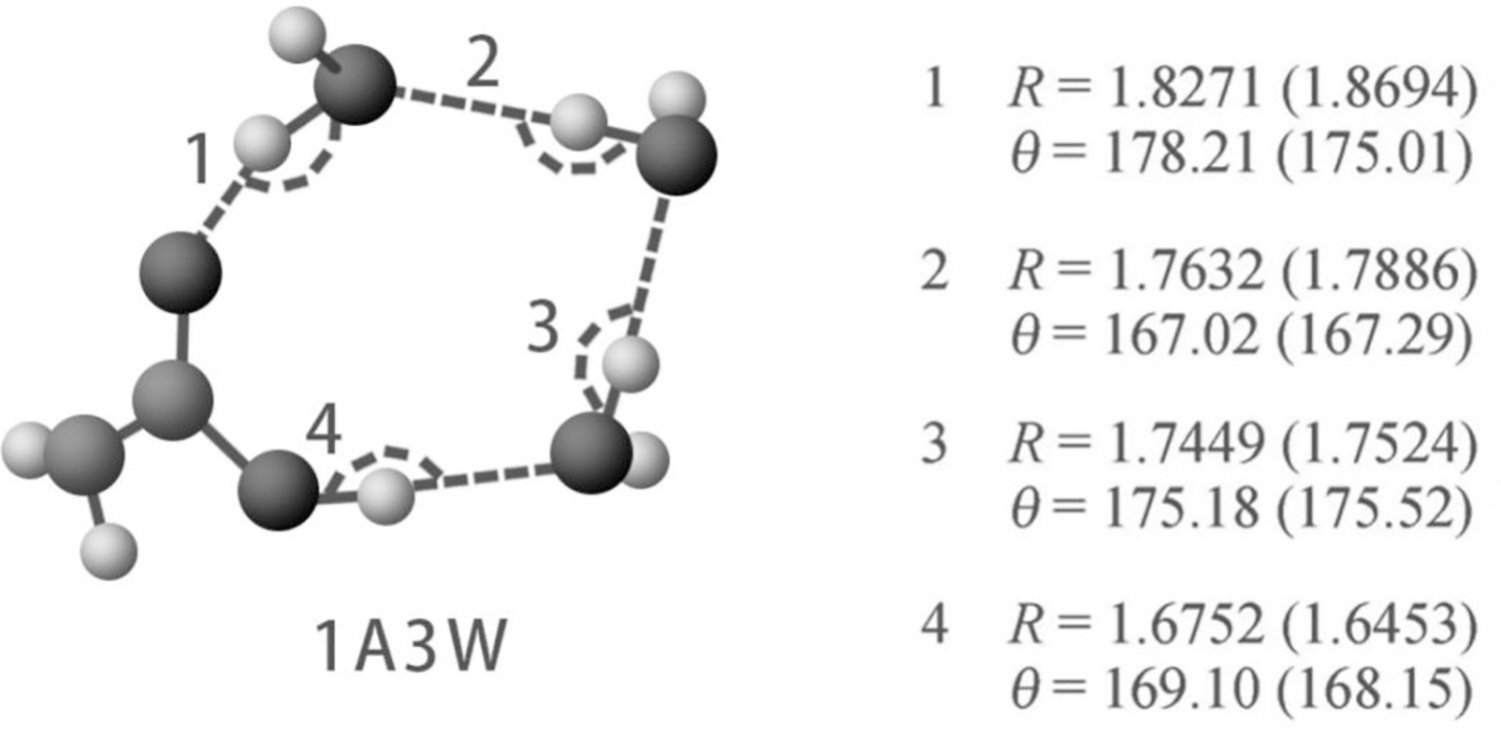
Equilibrium structure of a six-membered hydrogen-bonded ring containing a single acetic acid molecule and multiple water molecules. Hydrogen bonds are represented by dashed lines, with corresponding values listed nearby. Lengths (R) are in units of Å, and angles are in degrees. Data outside parentheses are values in the gas phase, while data inside parentheses are values in aqueous solution.

Since 2010, studies [29, 30] consistently show that in diluted acetic acid/water solutions, structures primarily form from complexes between acetic acid and water molecules The configurations and energies derived from quantum chemical calculations (QCC) within this investigation align closely with those observed in the gas phase. Expanding upon this research, we delved into the impact of solvent effects. QCC calculations were extended to encompass aqueous solutions, yielding geometric configurations, energies, and thermodynamic data pertaining to acetic acid/water aggregate structures in such solutions. Our findings reveal that in diluted acetic acid solutions (<10% acid content), six-membered hydrogen-bonded ring structures with individual acetic acid molecules are emerge, akin to those in spoiled rice wine. Rings with fewer than seventeen members exceed 1%, with five-membered and nine-membered rings each around 3% of the total. Refer to Fig 10 for details on the six-membered ring configurations.

Within this system, carbon atoms possessing moderate electronegativity and oxygen atoms exhibiting high electronegativity are arranged alternately, imparting explicithighly polar characteristic.To validate this hypothesis, we employ dipole moments to describe the overall deviation of a molecule from electrical neutrality due to an uneven distribution of charge within the molecule. Its calculation formula is outlined as follows:

p=q×d (Eq 3)

In the above equation, p represents the magnitude of the dipole moment, q represents the magnitude of the charge, and d represents the distance of charge distribution.

The orientation of the dipole moment coincides with the alignment of the charge distribution, and its magnitude scales directly with both the magnitude of the charge and the distance of the charge distribution, as determined by the computation method for the dipole moment.

The pertinent computations were conducted using Gaussian simulation software, yielding a dipole moment of 3.672 Debye under the B3LYP/def2-TZVPD basis set with dispersion function. In contrast, the experimentally determined average dipole moment for water stands at 1.85 Debye. This discrepancy underscores the heightened polarity and relative permittivity of acid-water cluster structures compared to clusters formed solely by water molecules. Consequently, these structures will exhibit elevated characteristics such as dielectric constant and dielectric loss.

## 5. Conclusion

In China, rice wine is typically stored in pottery jars [6], with a loss rate ranging from 5% to 10%. This study employs the polarization phenomenon under microwave conditions to assess the degree of deteriorated in rice wine, even in sealed containers.

The ensuing progress encompasses:

We found that distinct polarization behavior in deteriorated rice wine samples within high-frequency electromagnetic spectrum(specifically within the E-band microwave range of 60–69 GHz). As a validation, we use VNA to evaluate the dielectric parameters of diverse varieties and degrees of deterioration of rice wine both high and low-frequencies.

Subsequently, this study delved into examining the quantitative correlation between alterations in physicochemical parameters during the degradation of rice wine and the resultant high-frequency RCS signals.Established a comprehensive data system affirming the approximate linear correlation between fluctuations in total acidity and radar RCS. Further elaboration on the experimental data is provided in S2 Appendix. To elucidate the experimental findings, we put forward a ‘Hydrated Cluster hypothesis’, in consonance with experimental observations.

The insights gleaned from this research hold practical implications for the detection and mitigation of rice wine and wine deterioration during storage, particularly in large stainless-steel tanks [31], mitigating potential economic losses without opening the tanks. This research holds significance for brewing science beyond the realm of rice wine, as analogous degradation and aging phenomena occur in diverse liquid consumables like beer [32] and soy sauce [33, 34].

## Supporting information

S1 Appendix Correlation between radar imaging value Y and Radar Cross-Sections (RCS). (PDF)

S2 Appendix Total acidity analysis and comparative evaluation with RCS values. (PDF)
