# Supplementary material for: Discovery of electromagnetic polarization in Asian rice wine deterioration process and its applications
Source: PLoS One. 2024 Jun 20;19(6):e0302983. doi: 10.1371/journal.pone.0302983 (PMC11189232; doi:10.1371/journal.pone.0302983)
Supplement: S1 Appendix — (PDF) [file pone.0302983.s001.pdf]

S1 Appendix

Correlation between Radar Imaging Value Y and Radar Cross-Sections (RCS)

The quantified imaging value Y obtained directly from the millimeter-wave radar equipment is delineated as follows:

$$Y_{k-r} = \sum_{t=1}^{N_1} \sum_{r=1}^{N_2} \sum_{f} \frac{X_{f,t,r}}{g_{f,k,t,r}} \qquad (Eqn.S1)$$

Where Y_k denotes the received value at the target position k (target pixel), and $X_{f,t,r}$ represents the received frequency-domain signal corresponding to the transmission path from transmitting antenna t to receiving antenna r for electromagnetic waves with frequency f.

The received signal, denoted as $X_{f,t,r}$, encompasses the real and imaginary components of all signal points in the frequency domain, along with signals from every transmitting antenna frequency point. This implies that each transmitting antenna sequentially emits signals, ensuring the comprehensive data acquisition from all transmitting antennas.

$g_{f,k,t,r}$ symbolizes the frequency response from transmitting antenna t to receiving antenna r for position k during the transmission of electromagnetic waves at frequency f. The computation is articulated as follows:

$$g_{f,k,t,r} = \alpha_{f,k,t,r} \cdot e^{-j \cdot \omega_{f,k,t,r}} = \sqrt{\frac{G_t G_r \lambda_f{}^2 \sigma F_{k,t}{}^2 F_{k,r}{}^2}{(4\pi)^3 R_{k,t}{}^2 R_{k,r}{}^2}} e^{-j\frac{2\pi(R_{k,t}+R_{k,r})f}{c}} \qquad (Eqn.S2)$$

Wherein: G_t denotes the gain of the transmitting antenna, and G_r signifies the gain of the receiving antenna; in accordance with the analytical guidelines outlined in the radar instruction CD, the numerical values for G_t and G_r are both 5.8 dB.

λ_f represents the wavelength of the transmitted electromagnetic waves, contingent upon the frequency of the electromagnetic waves.

σ denotes the scattering cross-section, as prescribed by the analytical code detailed in the radar instruction CD; where $\sigma = 1$.

"c" represents the speed of light.

$R_{l,t}$ and $R_{k,t}$ denote the distances from the transmitting antenna "t" to the positions "l" and "k" of the scattering body, respectively.

$R_{l,r}$ and $R_{k,r}$ signify the distances from the receiving antenna "r" to the positions "l" and "k" of the scattering body, respectively.

$F_{l,t}$ and $F_{k,t}$ denote the propagation factors from the transmitting antenna "t" to the positions "l" and "k" of the scattering body, respectively.

$F_{l,r}$ and $F_{k,r}$ signify the propagation factors from the receiving antenna "r" to the positions "l" and "k" of the scattering body, respectively.

$$F_{k,t} = \sqrt{\cos^2 \frac{\theta_{k,t}}{2} (\sin^2 \varphi_{k,t} + \cos^2 \theta_{k,t} \cos^2 \varphi_{k,t})} \quad \textit{(Eqn.S3)}$$

$$F_{k,r} = \sqrt{\cos^2 \frac{\theta_{k,r}}{2} (\sin^2 \varphi_{k,r} + \cos^2 \theta_{k,r} \cos^2 \varphi_{k,r})} \qquad \textit{(Eqn.S4)}$$

Whereas: θ denotes the angle between the directional vector and the Z-axis; φ signifies the angle between the directional vector and the E-plane (XZ-plane).

The actual channel response encountered by the electromagnetic waves reflected at the given position shall be delineated as follows:

$$g_{f,l,t,r} = \alpha_{f,l,t,r} \cdot e^{-j \cdot \omega_{f,l,t,r}} = \sqrt{\frac{G_t G_r \lambda_f{}^2 \sigma F_{l,t}{}^2 F_{l,r}{}^2}{(4\pi)^3 R_{l,t}{}^2 R_{l,r}{}^2}} e^{-j \frac{2\pi (R_{l,t} + R_{l,r}) f}{c}} \qquad \textit{(Eqn.S5)}$$

Consequently, the matched filtering reception result at the computed position k (considering a single transmit-receive antenna pair, excluding $\sqrt{P_t}$) is determined as follows:

$$Y_{k-tr} = \sum_f \frac{X_{f,t,r}}{g_{f,k,t,r}} = \sum_f \frac{\left(\sum_{l=1}^{N_3} g_{f,l,t,r} + N_{f,t,r}\right)}{g_{f,k,t,r}} = \sum_f \left(\left(\sqrt{\sigma} + \sum_{\substack{l=1 \\ l \neq k}}^{N_3} \frac{g_{f,l,t,r}}{g_{f,k,t,r}} \right) + \frac{N_{f,t,r}}{g_{fk,t,r}} \right) \qquad \textit{(Eqn.S6)}$$

When an object is illuminated by electromagnetic waves, akin to the scenario depicted in Figure S1, energy scatters in all directions. The parameter that quantitatively defines the intensity of the scattered signal from the target is referred to as the Radar Cross Section (RCS), symbolized by the symbol σ.

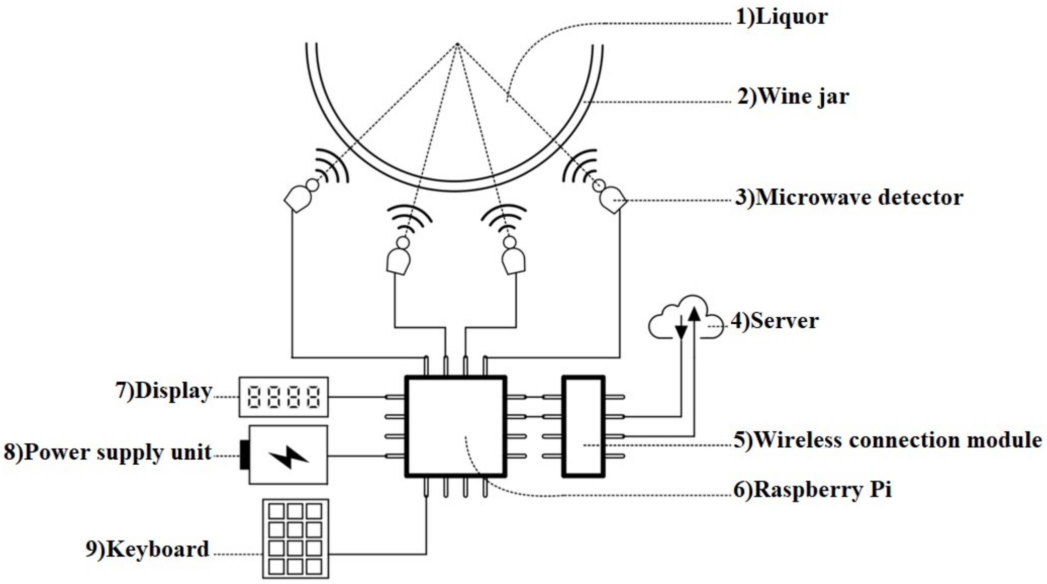

Fig S1.Schematic of the test. Where load 4 is the server(workstation)used to process the data uploaded by the microwave radar.

Assuming the RCS of the measured targets k_1 and k_2 are denoted as σ_1 and σ_2, respectively, under these experimental conditions, given that the radar front is positioned above the observed liquid in both cases and the measured targets reflect the surface, the ratio of their measurement results is approximately derived as follows:

$$r = \frac{(Y_{k1-tr})^2}{(Y_{k2-tr})^2} = \frac{\sigma_1}{\sigma_2} \quad \textit{(Eqn.S7)}$$

In simpler terms, the numerical ratio of the squared imaging value Y is directly proportional to the Radar Cross Section (RCS).
