# Supplementary material for: Discovery of electromagnetic polarization in Asian rice wine deterioration process and its applications
Source: PLoS One. 2024 Jun 20;19(6):e0302983. doi: 10.1371/journal.pone.0302983 (PMC11189232; doi:10.1371/journal.pone.0302983)
Supplement: S2 Appendix — (PDF) [file pone.0302983.s002.pdf]

S2 Appendix

Total Acidity Analysis and Comparative Evaluation with RCS Values

In order to ascertain the correlation between the total acid concentration in alcoholic beverages and the peak values detected by radar within the container, an additional experiment was devised by the research team. This experiment sought to examine the relationship between the total acid content of yellow rice wine and the radar RCS detection values, with the objective of analyzing the interplay between the molecular composition of liquid components and RCS values.

The initiation of degradation in rice wine involved the addition of 1g of yeast to 500ml of Shaoxing Jiafan rice wine, followed by placement in a constant temperature warm incubator box set at 37 °C. Subsequently, the total acidity and the corresponding radar cross-section (RCS) values of the rice wine were meticulously documented, with comprehensive data available in Table S-2.

Deterioration became detectable, and a sour taste emerged commencing from the third day. The mean total acidity for both the rice wine and the deteriorated rice wine group escalated to 9.90 g/L, accompanied by an elevation in the average radar-measured value compared to the initial measurements.

Sampling was conducted 2-3 times daily, with pre-test temperature adjustments to ensure uniformity. Each test, separated by at least one hour, spanned from July 9, 2023, to August 5, 2023, encompassing a 28-day period and yielding a comprehensive dataset of 94 sets.

Original data recorded in Table S2.

Table S2. Experimental data

Measure Date	Consumed sodium hydroxide (g)	Total acidity (g/L)	RCS Peak value	Liquid temperature (±0.5)° C
2023.7.9	7.1	6.3675	41141900	26
2023.7.10	7.15	6.4125	45801100	26
2023.7.11-10.00	8.05	7.2225	46052900	26
2023.7.11-16.00	7.95	7.1325	41011300	26
2023.7.11-20.35	8.05	7.2225	39827200	26
2023.7.12-13.00	7.85	7.0425	40130200	26
2023.7.12-14.00	8	7.1775	38173800	26
2023.7.12-15.00	7.95	7.1325	40130200	26
2023.7.12-16.00	8	7.1775	41359700	27
2023.7.12-17.00	8.1	7.2675	42365600	27.5
2023.7.12-21.00	8.15	7.3125	39151400	26
2023.7.13-11.00	7.95	7.1325	39021400	26
2023.7.13-13.00	8.1	7.2675	43310000	26
2023.7.13-14.00	8.15	7.3125	41750800	25
2023.7.13-15.00	8.15	7.3125	41022100	25.5
2023.7.13-16.00	8.15	7.3125	42207900	26
2023.7.13-17.00	8.2	7.3575	43977100	26.5
2023.7.13-20.00	8.1	7.2675	40897100	25
2023.7.14-12.45	8.15	7.3125	42561600	26.5
2023.7.14-14.00	8.2	7.3575	45267400	26

2023.7.14-15.00	8.25	7.4025	44867400	26
2023.7.14-16.00	8.3	7.4475	45092700	26
2023.7.14-17.00	8.3	7.4475	45773500	26
2023.7.14-20.00	8.2	7.3575	38720000	25
2023.7.14-20.30	8.2	7.3575	41179800	26
2023.7.14-21.00	8.1	7.2675	41325700	26.5
2023.7.15-13.30	8.2	7.3575	41213500	26
2023.7.15-14.30	8.2	7.3575	41062000	26
2023.7.15-15.30	8.15	7.3125	43983900	26.5
2023.7.15-16.30	8.25	7.4025	44238900	26.5
2023.7.15-17.30	8.45	7.5825	45954100	27
2023.7.15-19.30	8.1	7.2675	42445900	25.5
2023.7.15-20.30	8.15	7.3125	42589800	26
2023.7.16-14.00	8.3	7.4475	44220900	26
2023.7.16-15.00	8.5	7.6275	44763700	26
2023.7.16-16.00	8.55	7.6725	45636000	27
2023.7.16-17.00	8.7	7.8075	46601500	26
2023.7.16-19.00	8.4	7.5375	42272000	26
2023.7.16-20.00	8.5	7.6275	42525800	26
2023.7.16-21.00	8.5	7.6275	45563100	26.5
2023.7.17-14.00	8.55	7.6725	41182100	26
2023.7.17-15.00	8.6	7.7175	41603000	26
2023.7.17-16.00	8.6	7.7175	42074800	26
2023.7.17-17.00	8.65	7.7625	43458300	26
2023.7.17-19.00	8.55	7.6725	40460900	26

2023.7.17-20.00	8.6	7.7175	41121600	26.5
2023.7.17-21.00	8.5	7.6275	40824800	26
2023.7.19-14.00	8.85	7.9425	42916300	26
2023.7.19-15.00	8.75	7.8525	42819200	26
2023.7.19-16.00	8.75	7.8525	42028400	26
2023.7.19-17.00	8.8	7.8975	44734400	26
2023.7.19-18.00	8.8	7.8975	42434900	26
2023.7.19-19.00	8.8	7.8975	45254300	26
2023.7.19-20.00	8.8	7.8975	44570500	25
2023.7.21-15.00	8.7	7.8075	42354500	26
2023.7.21-16.00	8.7	7.8075	43304400	26
2023.7.21-17.00	8.6	7.7175	43627900	26.5
2023.7.21-18.00	8.6	7.7175	44133900	27
2023.7.21-19.00	8.6	7.7175	43832500	26
2023.7.21-20.00	8.6	7.7175	43632100	26
2023.7.22-16.00	8.65	7.7625	40141900	25
2023.7.22-17.00	8.65	7.7625	40313700	26
2023.7.23-15.00	8.4	7.5375	41396500	26
2023.7.23-16.00	8.45	7.5825	46322300	25.5
2023.7.24-14.00	8.4	7.5375	44834500	26
2023.7.24-15.00	8.4	7.5375	45091500	25
2023.7.25-17.30	8.75	7.8525	44043300	26
2023.7.26-15.00	8.6	7.7175	46929600	26
2023.7.26-16.00	8.55	7.6725	46174500	26
2023.7.27-15.00	8.25	7.4025	44781700	26

2023.7.27-16.00	8.5	7.6275	45475900	26
2023.7.28-14.00	8.6	7.7175	45420200	26
2023.7.28-15.00	8.6	7.7175	46678500	26
2023.7.29-15.00	8.75	7.8525	45564900	26
2023.7.29-16.00	8.85	7.9425	46297700	26
2023.7.30-16.00	8.7	7.8075	56615600	26
2023.7.30-17.00	8.85	7.9425	58879200	26
2023.7.30-18.30	8.65	7.7625	60610000	26
2023.7.30-20.00	8.8	7.8975	60776800	26
2023.7.31-14.00	8.7	7.8075	59974600	26
2023.7.31-15.30	8.85	7.9425	60113900	26
2023.7.31-17.00	8.7	7.8075	58095500	26
2023.8.1-19.30	8.95	8.0325	59122900	26
2023.8.2-15.00	9.2	8.2575	62052900	26
2023.8.2-16.30	8.85	7.9425	61081100	26
2023.8.2-18.30	8.75	7.8525	57191100	26
2023.8.3-16.30	8.7	7.8075	58047300	26
2023.8.3-19.00	8.8	7.8975	57261100	26
2023.8.4-15.00	8.75	7.8525	58608200	26
2023.8.4-17.00	8.8	7.8975	60668200	26
2023.8.4-19.00	8.75	7.8525	60430100	26
2023.8.5-15.00	8.75	7.8525	60689600	26
2023.8.5-17.00	8.8	7.8975	60335200	26
2023.8.5-19.00	9	8.0775	58545300	26

24
